# Who Needs Neighbors? *PKS8* Is a Stand-Alone Gene in *Fusarium graminearum* Responsible for Production of Gibepyrones and Prolipyrone B

**DOI:** 10.3390/molecules23092232

**Published:** 2018-09-02

**Authors:** Klaus Ringsborg Westphal, Asmus Toftkær Muurmann, Iben Engell Paulsen, Kim Tanja Hejselbak Nørgaard, Marie Lund Overgaard, Sebastian Mølvang Dall, Trine Aalborg, Reinhard Wimmer, Jens Laurids Sørensen, Teis Esben Sondergaard

**Affiliations:** 1 Department of Chemistry and Bioscience, Aalborg University, 9100 Aalborg, Denmark; kw@bio.aau.dk (K.R.W.); amuurm16@student.aau.dk (A.T.M.); ipauls16@student.aau.dk (I.E.P.); knorga15@student.aau.dk (K.T.H.N.); moverg16@student.aau.dk (M.L.O.); sdall16@student.aau.dk (S.M.D.); taalbo15@student.aau.dk (T.A.); rw@bio.aau.dk (R.W.); 2 Department of Chemistry and Bioscience, Aalborg University, 6700 Esbjerg, Denmark; jls@bio.aau.dk

**Keywords:** gibepyrones, prolipyrone, secondary metabolites, polyketide synthases, *Fusarium*

## Abstract

Genome sequencing of the genus *Fusarium* has revealed a great capacity for discovery of new natural products of potential economical and therapeutic importance. Several of these are unknown. In this study, we investigated the product of the *PKS8* gene in *Fusarium graminearum*, which was recently linked to gibepyrones in *F. fujikuroi*. Genomic analyses showed that *PKS8* constitutes a stand-alone gene in *F. graminearum* and related species. Overexpression of *PKS8* resulted in production of gibepyrones A, B, D, G and prolipyrone B, which could not be detected in the wild type strain. Our results suggest that *PKS8* produces the entry compound gibepyrone A, which is subsequently oxidized by one or several non-clustering cytochrome P450 monooxygenases ending with prolipyrone B.

## 1. Introduction

Filamentous fungi have been shown to produce a diverse array of secondary metabolites of therapeutic and economical importance [1,2,3]. Most of these secondary metabolites are produced by large enzyme complexes such as Non-Ribosomal Peptide Synthetases (NRPSs) and Polyketide Synthases (PKSs) [4]. As high amounts of genomic data have become available, the linking of biosynthetic genes to specific natural products has become an advancing field within genomics. The knowledge generated can be used to characterize new secondary metabolites and facilitates the prediction of the products of biosynthetic pathways [5]. Hansen et al. (2015) [6] showed that the genus *Fusarium* contains several gene clusters containing *PKSs* and *NRPSs* and the genus is thus a rich source for the discovery of new bioactive compounds. Sequencing of the grain crop pathogenic fungus *F. graminearum* [7,8] revealed that the filamentous fungus’ genome encodes 16 *PKSs* but only eight of these have been linked to a specific product [6]. One of the *PKSs* for which the product remains to be identified is *PKS8* (Hansen et al. 2012 [9] numeration), which is a reducing iterative type I PKS containing the following domains: a keto-synthase, an acetyltransferase, a dehydratase, a methyltransferase, an enoylreductase, a ketoreductase and an acyl carrier protein [6,10]. An orthologue of *PKS8* was recently linked to biosynthesis of the α-pyrones gibepyrone A–F in *F. fujikuroi* [10]. In *F. fujikuroi*, *PKS8* (GPY1; FFUJ_12020) is located in a two-gene cluster together with an ABC transporter (GPY2; FFUJ_12021), which has some influence on efflux of gibepyrones out of the fungal cells [10].

**Figure 1:**
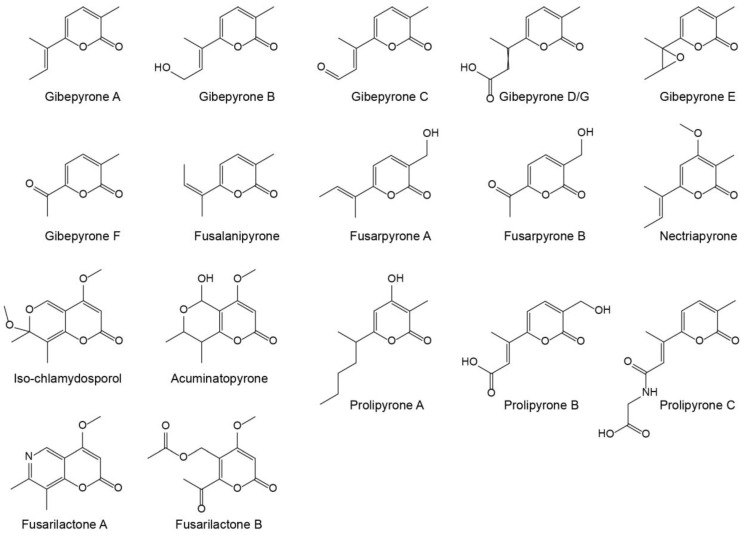
Structures of known α-pyrones produced by *Fusarium*.

Gibepyrones have previously been detected in several *Fusarium* species, including *F. fujikuroi* [11], but not in *F. graminearum*. Several additional α-pyrones have also been identified in *Fusarium* (Figure 1), of which some potentially could originate from the same biosynthetic pathway as gibepyrones. 

Since the *PKS8* in *F. graminearum* potentially could be responsible for producing novel α-pyrones the aim of this study was to identify the products derived from this gene cluster.

## 2. Results and Discussion

### 2.1. Comparison of the PKS8 Gene Cluster

**Figure 2:**
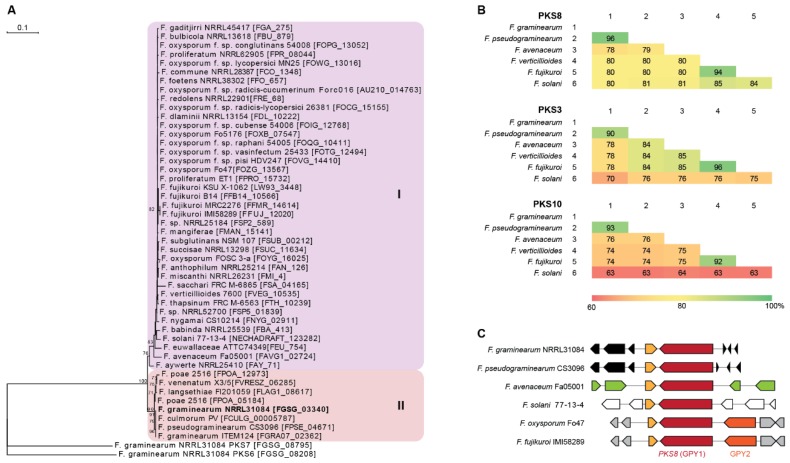
Comparison of PKS8 and the gibepyrones biosynthesis gene clusters. (**A**) Maximum parsimony tree based on amino acid sequenced of the KS domains from *Fusarium* PKS8 orthologues with *F. graminearum* PKS6 and PKS7 as outgroup. Numbers represent percentage bootstrap support (>65) from 100 replications. (**B**) Similarity matrices (% identity) of the amino acid sequences of the six PKS8, PKS3 and PKS10 orthologues based on clustalW alignments. (**C**) Overview of *PKS8* (*GPY1*) and neighboring genes in *F. graminearum* (FGSG_03344-03338), *F. pseudograminearum* (FPSE_04677-04668), *F. avenaceum* (FAVG1_02827-22), *F. solani* (NECHADRAFT_30741-99039), *F. oxysporum* (FOZG_13564-13570) and *F. fujikuroi* (FFUJ_12017-12024).

*PKS8* is very conserved throughout the *Fusarium* genus as it is one of only three *PKSs* present in all 31 genome sequenced *Fusarium* species analyzed by Brown and Proctor [12]. Construction of a phylogenetic tree based on 47 PKS8 orthologues from *Fusarium* resulted in two major clades (Figure 2A), where PKS8 from *F. graminearum* was located in clade II together with orthologues from other species also belonging to the *sambucinum* species complex [13]. This is also reflected in comparison of the entire PKS8 between the selected strains, where the sequence identity of *F. graminearum* PKS8 was 96% on amino acid level compared to the orthologue in *F. pseudograminearum* and 78–80% to *F. avenaceum*, *F. verticillioides*, *F. fujikuroi* and *F. solani* (Figure 2B). This is a relatively high sequence identity compared to the two other widely distributed PKSs with known products in *Fusarium*; PKS3 (fusarubins; [14]) and PKS10 (fusarins; [15]), where sequence identities of 70–90% and 63–93%, respectively, were observed.

Examination of the genes surrounding *PKS8* showed that the ABC transporter (GPY2) is present in *F. fujikuroi*, where deletion resulted in significantly enhanced level of intracellular gibepyrone A [10]. The gene is also present in closely related species such as *F. verticillioides* and *F. oxysporum*, being absent in *F. graminearum*, *F. pseudograminearum*, *F. avenaceum* and *F. solani* (Figure 2C). However, analyses of the clusters of the strains located in clade I showed that the ABC transporter is not located next to *PKS8*. The most similar ABC transporter in *F. graminearum* (FGSG_02316) shares 31% sequence identity on amino acid level and is located on chromosome 1, instead of chromosome 2. This gene is located next to *NRPS4*, which is responsible for production of a yet unidentified surface hydrophobicity increasing non-ribosomal peptide [16]. 

A putative *S*-adenosylmethionine-dependent methyltransferase is located immediately downstream of *PKS8* in *F. graminearum* (FGSG_03341) and in all of the other examined species. Gibepyrone production was however not influenced by deletion of this gene in *F. fujikuroi* and the methyltransferase domain of PKS8 is therefore hypothesized to be responsible for addition of the two methyl-groups in gibepyrone. Thus, the independent methyltransferase is not regarded as part of the gene cluster [10]. Together, these observations suggest that *PKS8* constitutes a solitary gene cluster in *F. graminearum* and related species.

### 2.2. Identification of the Products from PKS8

**Figure 3:**
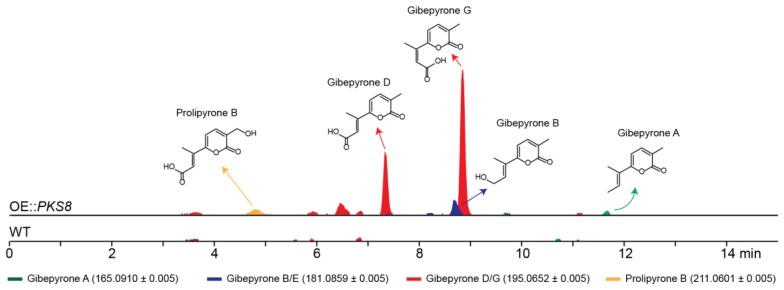
Chemical analyses of the *F. graminearum* OE::*PKS8* and wild type strains showing the extracted chromatograms for the protonated ions of the tentatively detected α-pyrones.

To identify the products of *PKS8* in *F. graminearum* a constitutive promoter was introduced in front of the gene. Five strains were verified by PCR and the metabolite profiles of two of these strains were subsequently analyzed. A preliminary study showed that both strains had similar metabolite profiles and therefore one strain was selected for further analyses. The production of secondary metabolites in one of the resulting mutants (OE::*PKS8*) was examined by HPLC coupled to a high-resolution mass spectrometer (HRMS) and compared to the wild type (Figure 3). Using a dereplication approach applying a database of known *Fusarium* α-pyrones, we tentatively identified major peaks corresponding to gibepyrone D and G. These compounds are *E*-*Z* isomers, and thus have identical masses ([M + H]^+^ = 195.0652 Da), which can make HRMS based identification difficult (Figure S1). However, as gibepyrone D has been found to elute before gibepyrone G in reverse-phase HPLC [17], we assumed that the compound eluting after 7.3 min was gibepyrone D and that gibepyrone G is the metabolite eluting at 8.9 min. Through dereplication, we also identified three minor peaks matching prolipyrone B ([M + H]^+^ = 211.0601) as well as gibepyrone A ([M + H]^+^ = 165.0910) and B ([M + H]^+^ = 181.0859) eluting after 4.9, 11.7 and 8.7 min, respectively. Prolipyrone B has so far only been identified in a *F. proliferatum* strain together with gibepyrone D [18]. The gibepyrones and prolipyrone B observed in the OE::*PKS8* mutant were not present in the wild type strain, which suggests that the compounds are derived from the same biosynthetic pathway that has been activated through overexpression of the *PKS* gene. 

To verify the identity of some of the metabolites observed in the HRMS analyses, we isolated prolipyrone B and gibepyrone D through preparative HPLC and analyzed by ^1^H-NMR (Figures S2 and S3). For both compounds the resulting ^1^H-NMR data (Table 1) were in agreement with reported spectra of [17,18,19] and thereby confirming their identity.

### 2.3. Biosynthesis of Gibepyrones in F. graminearum

**Figure 4:**
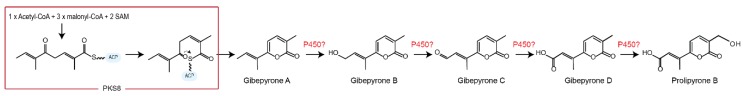
Proposed biosynthetic pathway of gibepyrones starting with production of gibepyrone A and ending with prolipyrone B through oxygenation by one or several non-clustering cytochrome P450 monooxygenases.

Janevska et al. 2016 [10] showed that gibepyrones B and D were derived from gibepyrone A, the primary polyketide product of *F. fujikuroi PKS8*, through oxidative activity by one or more non-cluster related cytochrome P450 monooxygenases (Figure 4).

Gibepyrone C, which represents the intermediate between gibepyrones B and D was not observed in our present study or in *F. fujikuroi* [10], suggesting that this molecule is quickly processed to gibepyrone D. Based on the chemical structures of the observed compounds, we suggest that prolipyrone B is derived through additional oxygenation of gibepyrone D by an additional cytochrome P450 monooxygenase.

**Figure 5:**
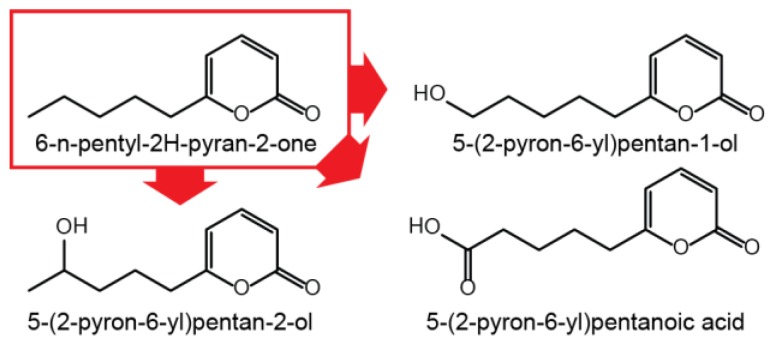
Oxidation of 6-n-pentyl-2H-pyran-2-one in *F. graminearum*.

Since gibepyrone A has a moderately toxic effect on the fungus itself, oxidation of gibepyrone A to its derivatives gibepyrones B and D by non-cluster related enzymatic activity could be a mechanism of detoxification [10]. A mechanism analogous to this has been observed for the conversion of toxic 6-pentyl-2H-pyran-2-one to less toxic derivatives trough oxidation in *F. graminearum* (Figure 5) [20] and for the conversion of toxic fusaric acid by cytochrome P450 monooxygenases in *F. fujikuroi* [21].

Since no cluster-related P450 cytochrome monooxygenase was found in *F. graminearum*, the conversion of gibepyrone A to gibepyrone D could be the result of an applied detoxification mechanism in response to an ineffective secretion pathway for gibepyrone A. This could explain that only a minor peak corresponding to gibepyrone A was detected in the OE::*PKS8* mutant.

## 3. Materials and Methods

### 3.1. Bioinformatics

*PKS8* orthologues were identified in GenBank through BlastP analyses using orthologues from *F. graminearum* (FGSG_03340) and *F. fujikuroi* (FFUJ_12020) as query. The KS domains from the resulting 47 *PKS8* orthologues were extracted as previously described [6,9]. Analyses of the KS domains was performed in CLC Main Workbench 8.0.1 (Qiagen, Hilden, Germany) where a maximum likelihood phylogenetic (PHYML) tree was constructed using 100 bootstrap replications and PKS7 (FGSG_08795) and PKS6 (FGSG_08208) from *F. graminearum* as outgroup. Selected full-length *PKS8*, PKS3, and PKS10 encoded proteins from *F. graminearum*, *F. pseudograminearum*, *F. avenaceum*, *F. verticillioides*, *F. fujikuroi* and *F. solani* were subjected to pairwise comparison as previously described [22].

### 3.2. Overexpression of PKS8 in F. graminearum

The flanking regions of *PKS8* was PCR amplified from *F. graminearum* PH-1 (NRRL31084) using primers *PKS8*-O1 to *PKS8*-O4 listed in Supplementary (Table S1) and Pfu polymerase (Stratagene, La Jolla, CA, USA). The PCR fragments were cloned into a linearized pRF-HU2E vector under control of the gpdA promoter by a four fragment cloning step using the USER enzyme™ (New England Biolabs, Ipswich, MA, USA) and verified by colony PCR [23]. Transformation of *F. graminearum* was carried out by *Agrobacterium tumefaciens* mediated transformation as described previously [24] and the resulting mutants were verified by diagnostic PCR using a forward primer annealing to gDNA outside the border region and a reverse primer annealing to the hygromycin resistance gene.

### 3.3. Chemical Analyses

Wild type *F. graminearum* (PH-1) and OE::*PKS8* were prior to the experiment grown for one week in the dark at 25 °C on solid yeast extract sucrose (YES) medium in 90 mm petri dishes [25]. Mycelium from the strains were transferred to new solid YES plates by three-point inoculation in triplicates and cultivated for two weeks. Secondary metabolites were extracted based on Smedsgaard 1997 [26], where nine plugs (8 mm) were extracted with 3 mL ethyl acetate:dichloromethane:methanol (3:2:1) with 1% formic acid in ultrasonic bath for 45 min. The extracts were transferred to a new tube and evaporated under a stream of nitrogen. The extracts were redissolved in 600 µL methanol spun in centrifuge tubes for three min at 14.1 rcf to remove impurities before the extracts were transferred to 2 mL HPLC vials. 

The samples were analyzed by high performance liquid chromatography (Hitachi Elite LaChrom HPLC, Hitachi, Tokyo, Japan) coupled to a high-resolution mass spectrometer (HRMS; Bruker compact MS ESI-Q-TOF, Bruker Daltonics, Bremen, Germany) by a 5:95% flowsplitter. 10 µL extracts were separated on a C18 column (Ascentis Express C18, 15 cm × 4.6 mm, 2.7 µm pore size, Sigma-Aldritch, St. Louis, MO, USA) at 40 °C using a 1 mL/min flow and a gradient system consisting of water and acetonitrile both with 0.1% formic acid. The gradient started at 10% acetonitrile increasing to 100% over 20 min and maintaining at 100% for further 10 min. A database of known α-pyrones from *Fusarium* consisting of gibepyrone A–G, fusalanipyrone, fusarpyrone A and B, nectriapyrone, chlamydosporols, acuminatopyrone, prolipyrone A–C and fusarilactone A was constructed. This database was used for dereplication using the protonated ions as previously described [27].

### 3.4. Isolation and Structural Elucidation of Prolipyrone B and Gibepyrone D

For large-scale extraction, the OE::*PKS8* mutant was grown on 68 solid YES plates (85 mm) were three-point inoculated and cultured for two weeks in the dark at 25 °C. After cultivation the plates were sliced into cubes of approx. 5 × 5 mm and extracted ultrasonically with sufficient ethyl acetate:dichloromethane:methanol (3:2:1) with 1% formic acid to cover the agar cubes. The extract was filtered through MiraCloth (Calbiochem, Merck, Darmstadt, Germany), evaporated to dryness in a rotary evaporator at 40 °C and subsequently lyophilized. The oily crude extract was redissolved in a total of 7 mL methanol and phase separated by spinning in centrifuge tubes for 6 min at 14.1 rcf. The methanol (top) phases were transferred to a 12 mL tube, nitrogen evaporated to approx. 3 mL and 2.5 mL were transferred to two 2 mL HPLC-vials.

The extract was run on an Agilent 1260 infinity semi-preparative HPLC system using a DAD VL detector (Agilent Technologies, Santa Clara, CA, USA). 22 runs of 100 µL were injected and separated on a Luna^®^ C18 LC column (5 µm, 250 × 10 mm, Phenomenex, Torrence, CA, USA) using a flow of 5 mL/min and a gradient system of A (water with 0.05% TFA) and B (acetonitrile with 0.05% TFA). The gradient started with 10% B increasing to 100% in 12 min and was maintained for 2 min before reversion to 10% over 2 min and recalibration for 6 min. Peaks containing prolipyrone B and gibepyrone D were isolated, lyophilized and re-dissolved in 550 µL DMSO-*d*_6_ or 6000 µL CD_3_OD, respectively, both supplemented with 0.03% TMS. The extracts were analyzed with ^1^H-NMR on a Bruker AVIII-600 MHz NMR spectrometer at either 50 °C for prolypyrone B or 25 °C for gibepyrone D. 

## Figures and Tables

**Table 1:** Structure, with important hydrogen couplings, and ^1^H-NMR spectroscopic data (600 MHz) of gibepyrone D and prolipyrone B.

	**Gibepyrone D (CD_3_OD)**		**Prolipyrone B (DMSO-*d*_6_)**		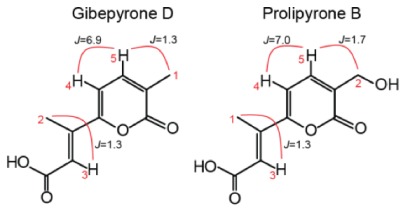
	**type**	**δ_H_ (*J* in Hz)**	**Type**	**δ_H_ (*J* in Hz)**	
1	CH_3_	2.11 (d, 1.3)	CH_3_	2.31 (d, 1.3)	
2	CH_3_	2.36 (d, 1.3)	CH_2_	4.30 (s, brd)	
3	CH	6.61 (q, 1.3)	CH	6.45 (q, 1.3)	
4	CH	6.70 (d, 6.9)	CH	6.85 (d, 7.0)	
5	CH	7.39 (dq, 1.3; 7.0)	CH	7.52 (dt, 1.7; 7.0)	

## Supplementary Materials

The supplementary materials are available online.
