# Supplementary material for: Who Needs Neighbors? PKS8 Is a Stand-Alone Gene in Fusarium graminearum Responsible for Production of Gibepyrones and Prolipyrone B
Source: Molecules. 2018 Sep 2;23(9):2232. doi: 10.3390/molecules23092232 (PMC6225250; doi:10.3390/molecules23092232)
Supplement: Supplementary file 1 [file molecules-23-02232-s001.pdf]

Supplementary table 1. Primers used in the experiments

Primer name	Sequence 5´→ 3´	Function
PKS8-O1	GGTCTTAAUGGATCTAATTCTAGACCCCTGCATGG	Overexpression
PKS8-O2	GGCATTAAUGTGTGATACAGGATGAACTGAACAGAACA	Overexpression
PKS8-O3	GGACTTAAUGCCTTCTCAAATTCAACCATGGC	Overexpression
PKS8-O4	GGGTTTAAUATCGTTAGCTATACATGATGGTATGTGTGG	Overexpression
PKS8-V1	CCTGGTATGAAATGATTAGCGGAGT	Verification of mutants
PKS8-V2	CTTTCACAAAGTGTGCACCTCTGAA	Verification of mutants
PKS8-V3	ATGGTGACCTCACACGAGTGGC	Verification of mutants
PKS8-V4	AACAACGGCGTGCGCAGTTGAT	Verification of mutants
HYG-58-1	AGCTGCGCCGATGGTTTCTACAA	Verification of mutants
HYG-58-2	GCGCGTCTGCTGCTCCATACAA	Verification of mutants

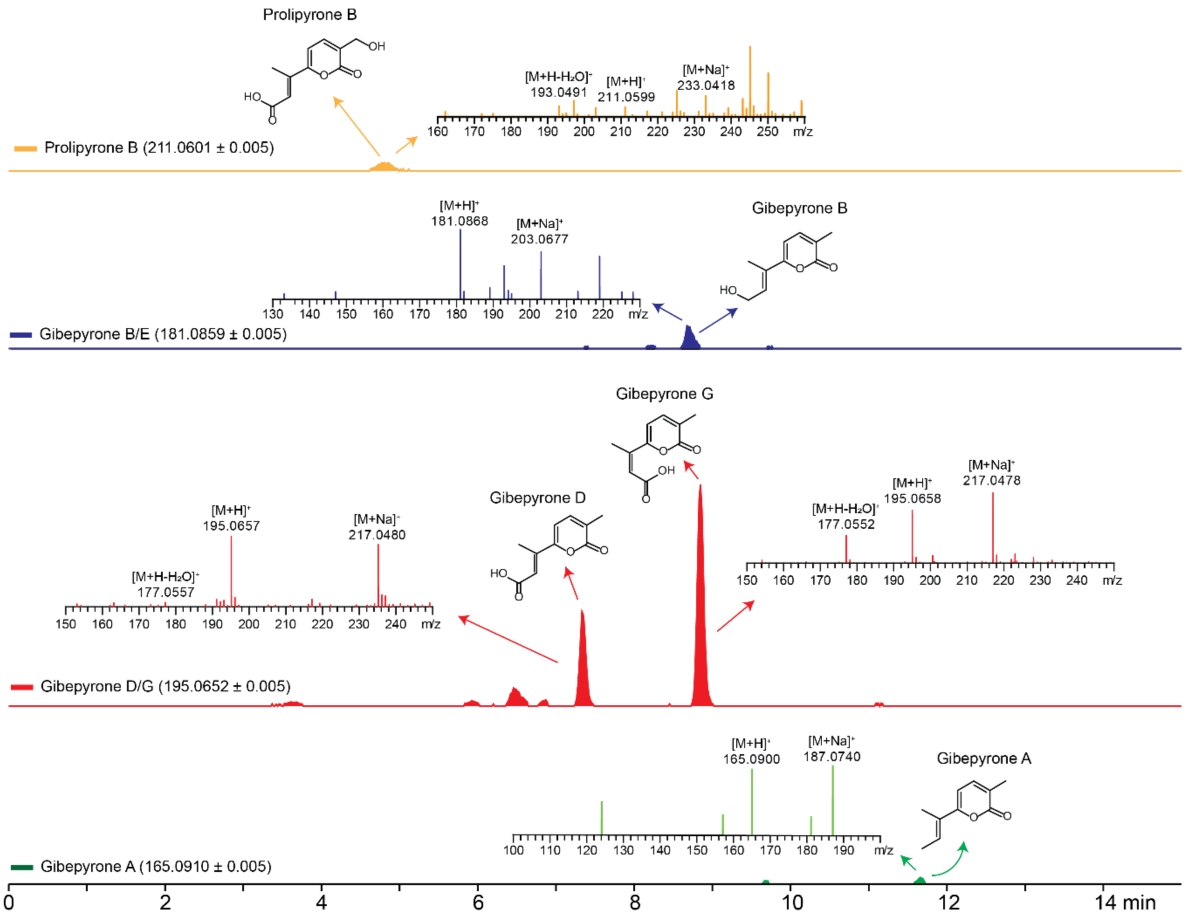

Supplementary figure 1. Extracted ion chromatograms for the protonated ions [M+H]+ of gibepyrone A, B, D, E and prolipyre B with inserted mass spectra for the individual peaks.

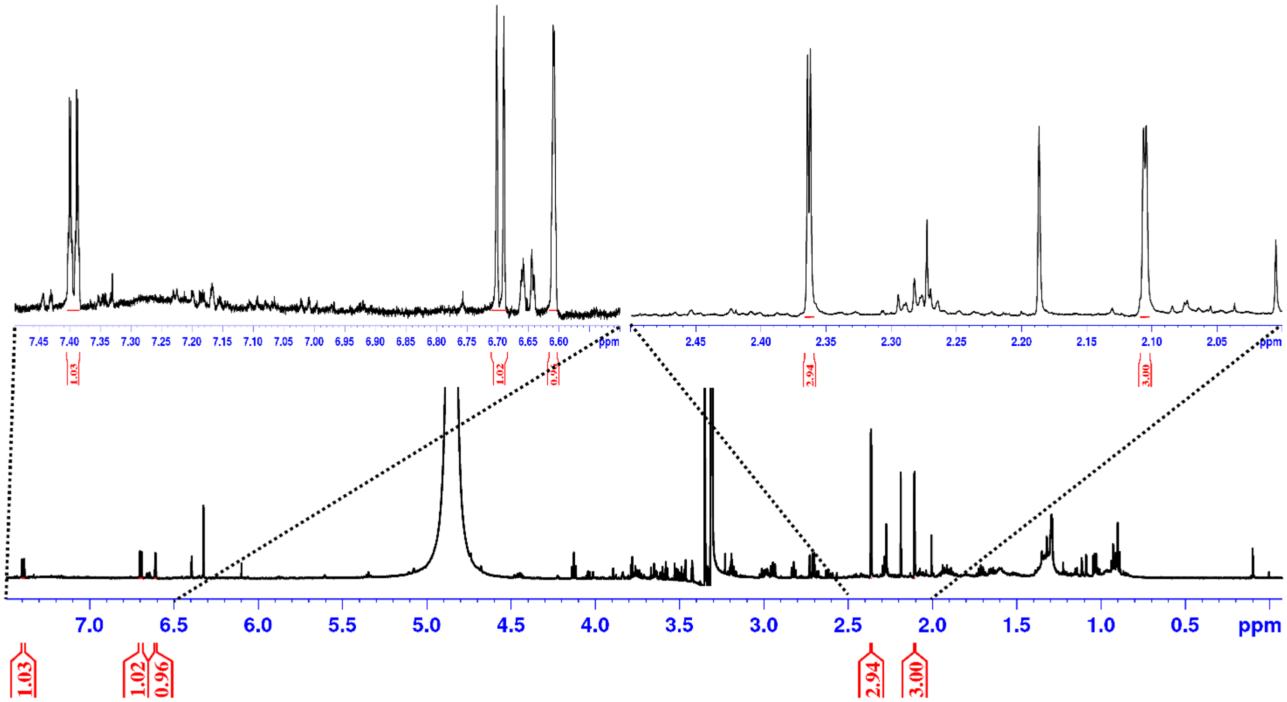

Supplemental figure 2: 1H NMR spectrum of gibepyronD (600 MHz, 25°C, CD_3OD) including two magnifications over regions of interest. Integrals are shown in red calibrated to 3.00 H for the methyl group at 2.11 ppm. Axis calibrated by TMS =0.

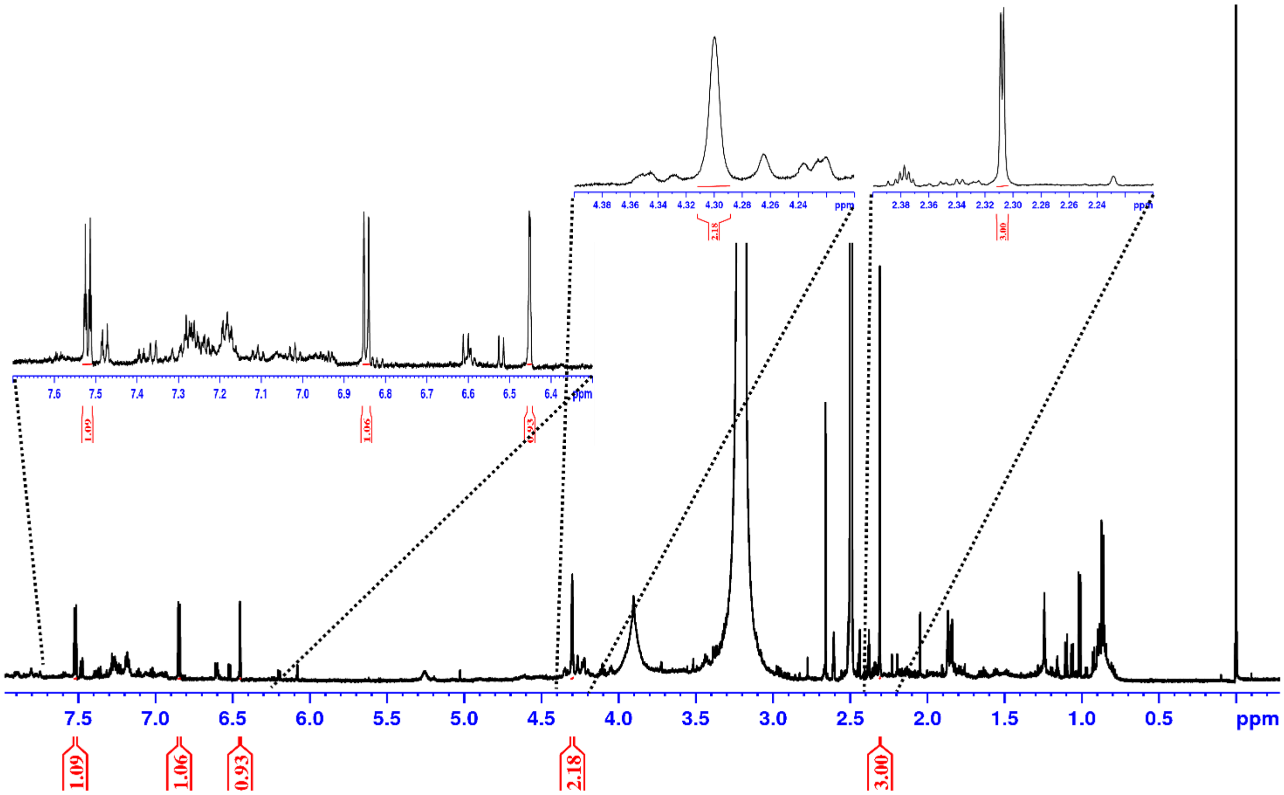

Supplemental figure 3: 1H NMR spectrum of prolipyrone B (600 MHz, 50°C, DMSO-d6) including three magnifications over regions of interest. Integrals are shown in red calibrated to 3.00 H for the methyl group at 2.31 ppm. Axis calibrated by TMS =0.
